# A Complete Closure of Hymen

**Published:** 1874-06

**Authors:** G. C. Paoli


ARTICLE IV.—*A Complete Closure of Hymen.* By G. C. PAOLI, M.D.

On the 10th of February Dr. S. called at my residence, and requested me to go with him to see a girl fifteen years of age, who he said was suffering intense pains from a tumor of the uterus. As the doctor had no idea of the character of the tumor, he applied leeches to subdue the inflammation.

On my arrival I found the patient was in agony from excruciating pains in the back and abdomen. Her face was in cold, clammy perspiration; her hands were cold; pulse 120, and weak; abdomen somewhat distended, and tender to the touch. By digital examination I found that when my finger reached the inside of the vulva it came upon a very tense, elastic swelling. By ocular examination I found that said tumor protruded like a sack, and at its centre it was so thin and shining that I could perceive it contained a dark fluid. Satisfied that the case was a complete closure of hymen, I ruptured it at once with my finger, and about three quarts of menstrual fluid were evacuated, and the girl is doing well.

But as simple and plain as this case seems to us, there are still many cases on record which have proved fatal after the perforation of the membrane for the relief of menstrual retention. Blood has been sometimes effused into the peritoneal cavity. In other cases death has occurred from peritonitis and pyemia.

